# KinoViz: A User-Friendly Web Application for High-Throughput Kinome Profiling Analysis and Visualization in Cancer Research

**DOI:** 10.21203/rs.3.rs-6431257/v1

**Published:** 2025-06-30

**Authors:** Ehsan Saghapour, Joshua C. Anderson, Jake Y. Chen, Christopher D. Willey

**Affiliations:** The University of Alabama at Birmingham; The University of Alabama at Birmingham; The University of Alabama at Birmingham; The University of Alabama at Birmingham

**Keywords:** Kinomics, Glioblastoma, Ex Vivo Profling, Patient-derived xenografts

## Abstract

Kinases, at the signaling level, dynamically mediate uncontrolled cellular growth, survival and other cancer supporting processes. This, paired with the inherent druggability of kinases, points to the importance of measuring kinase activity, and that of inhibitors against them, directly, and to analyze this accurately. High-throughput kinome profiling technologies, such as the PamStation^®^12, allow researchers to kinetically capture kinase activity, against a multitude of peptide targets simultaneously. Yet, the complex datasets produced often require advanced computational tools and bioinformatics expertise to properly analyze that are not intuitive or readily available. To address this gap, we developed KinoViz, a web-based application to simplify analysis and visualization of kinome array data. KinoViz offers a suite of interactive tools that enables users to upload raw peptide phosphorylation datasets and conduct in-depth analyses without the need for coding knowledge. Key features include modules for visualizing kinetic phosphorylation curves, identifying statistically significant peptide changes, exploring individual peptide profiles, and generating insightful visualizations such as heatmaps, network diagrams, and dimensionality reduction plots (PCA, UMAP). By making complex kinomic data more accessible and interpretable, KinoViz allows researchers to rapidly generate interactive visualizations and comparative analyses. We aim to expand KinoViz’s analytical capabilities for more advanced use, including use in direct translational drug discovery.

## 1. Introduction

Cancer often involves dysregulated kinase signaling, driving abnormal cell growth and survival. The human kinome comprises over 500 kinases that regulate critical processes (proliferation, apoptosis, metabolism), and many oncogenic mutations or aberrations occur in kinase pathways[1, 2]. Accordingly, protein kinases have become key therapeutic targets in oncology, with numerous kinase inhibitors in clinical use or development[3, 4]. Accurately identifying which kinases are abnormally active in a tumor can properly guide targeted therapies and improve understanding of tumor biology.

Array-based kinome profiling platforms enable high-throughput measurement of kinase activity in biological samples. One such technology, the PamStation^®^12 (PamGene), utilizes microarrays with peptide targets (PamChips^®^) to measure real-time phosphorylation events. Each PamChip contains hundreds of different immobilized peptide substrates derived from known phosphorylation sites on proteins, that collectively represent a broad portion of the kinome (tyrosine ‘Y’ kinome or serine ‘S’/threonine ‘T’ kinome depending on chip type). Repeated pumping of cellular or tissue lysates across the array of phosphorylatable peptide targets allows kinetic measurement of active kinases[5, 6].Fluorescently labeled (FITC) anti-phospho-residue (Y, S or T) specific antibodies detect phosphorylation of these peptides with charge-coupled device (CCD) image capture as often as every few seconds. This generates a rich dataset including multiple time points (reaction cycles), with multiple exposures for each peptide target. The resulting signal traces reflect the enzymatic activity of upstream kinases present in the sample[7]. Analyzing specific peptide phosphorylation in a kinetic manner during the reaction and at steady state (i.e., end level) helps determine relative specific kinase activity. In other words, PamChip kinome profiling surveys the activity of many kinases simultaneously within a sample and is, thus, an important tool in cancer research and drug discovery.

Kinome profiling utilizing peptide arrays has been utilized in a multitude of cancers, identifying signaling, molecular targets, subtypes, resistance mechanisms, as well as direct drug response[7–13]. Despite its value, kinome array data are complex to analyze. Raw images require processing including initial image gridding, signal with background subtraction, scaling/normalization, and statistical comparison. While PamGene’s proprietary BioNavigator software can perform data processing along with an *Upstream Kinase Analysis* (UKA) to predict likely responsible kinases, extracting biological insights often demands custom analyses and visualization. Researchers have developed bioinformatic pipelines (e.g. KRSA, Kinome Random Sampling Analyzer R Shiny app) to interpret kinome data and predict active kinases [2, 7, 14–16]. However, using such tools can require computational expertise or intensive manual steps. As the adoption of high-throughput kinome profiling increases, there is a need for user-friendly software to streamline the analysis and visualization of these data[14]. In particular, interactive web applications can improve reproducibility and accessibility by enabling bench scientists to explore their kinome results quickly, identifying key peptides/kinases, and generate publication-ready figures without writing code.

To address this need, we developed KinoViz, a web application for kinome array data analysis and visualization. The application provides an intuitive interface for oncology researchers (and other biomedical scientists) to upload kinome profiling data and interactively explore phosphorylation patterns. Key modules within KinoViz allow users to: (1) view a global “peptide landscape” of the kinome activity, (2) examine kinetic phosphorylation curves, (3) pinpoint the most important peptide changes between experimental conditions, (4) analyze individual peptide data in detail, and (5) visualize data for pattern recognition or dimensionality reduction (i.e., heatmaps, PCA). In this paper, we describe the development and features of the KinoViz application and demonstrate its utility using a kinome profiling case study in patient-derived xenograft (PDX) tissue lysates from glioblastoma (GBM). We show that KinoViz can effectively handle PamStation data, highlighting drug target effects *ex vivo*. The results underscore the application’s relevance for oncology discovery research, providing a new tool to rapidly interpret kinome activity profiles.

## 2. Materials and Methods

The web application leverages the Shiny framework[17] from the R ecosystem and integrates multiple packages to enhance functionality and user experience. We designed the layout and dashboards using *shinydashboard*[18] with styling from shinythemes[19], while interactive data tables rely on *DT*[20]. Data input and manipulation are executed with *readr*[21], *data.table*[22], and *dplyr*[23]. For graphical representations, we employ *ggplot2*[24], *plotly*[25], *heatmaply*[26], and *ggridges*[27]. Network analyses use *igraph*[28], and data reshaping is performed by *reshape2*[29] and *tidyr*[30]. Additionally, advanced interactivity is achieved with *aggrid* and *jsonlite*[31], and we use the *umap* package[32] for dimensionality reduction.

Samples analyzed for this study include 27 human-derived GBM PDX tumors heterotopically passaged in athymic nude mice under an approved animal protocol (UAB IACUC-21435), though they can be implanted orthotopically in the brain (Supplemental table 1)[33–36]. These tumors were provided by the UAB Brain Biorepository/Animal Models Core as deidentified samples allowing for a waiver of informed consent and HIPAA with Institutional Review Board Approval (IRB-300002910). Some of these tumors had acquired standard of care therapeutic resistance to either temozolomide (TMZ) or fractionated radiation treatment (RT) through serial treatment as previously published[33, 34]. Heterotopicallyimplanted tumor tissue was surgically extracted from mice as described previously, prior to lysis in MPER buffer (Thermo Fischer Scientific, Waltham, MA), prior to protein quantification. *Ex vivo* drug testing was conducted in previously lysed PDX tissues (15μg of protein in 25 μl), that had either DMSO vehicle control, 5 μM brigatinib, sitravatinib, or neratinib “spiked in” for 15 minutes at 4C, prior to loading onto the tyrosine kinase specific PamChip. These drugs were purchased from Selleckchem (Houston, TX).

We conducted kinomic profiling of the above-mentioned samples on the PamStation^®^12 platform (PamGene International, The Netherlands), which facilitates high-content kinase activity analysis via peptide microarrays using standard practices within the UAB Kinome Core[34, 37–40]. Lysates, along with buffers including ATP and FITC-conjugated anti-phospho-tyrosine antibodies, were applied to PamChips (PTK article number 86402) containing immobilized peptide substrates printed upon a porous aluminum-oxide surface[33]. Real-time FITC (CCD) imaging of peptide phosphorylation was captured over ~ 80 minutes, spanning several cycles and exposure times. Following image capture, BioNavigator (v6.3) software processed the raw images, locationally identifying and gridding ‘spots’, subtracting background noise, and quantifying intensities [33]. Signal minus background was then exported as CSV files for further analysis in KinoViz.

## 3. KinoViz Overview

**Figure 1:**
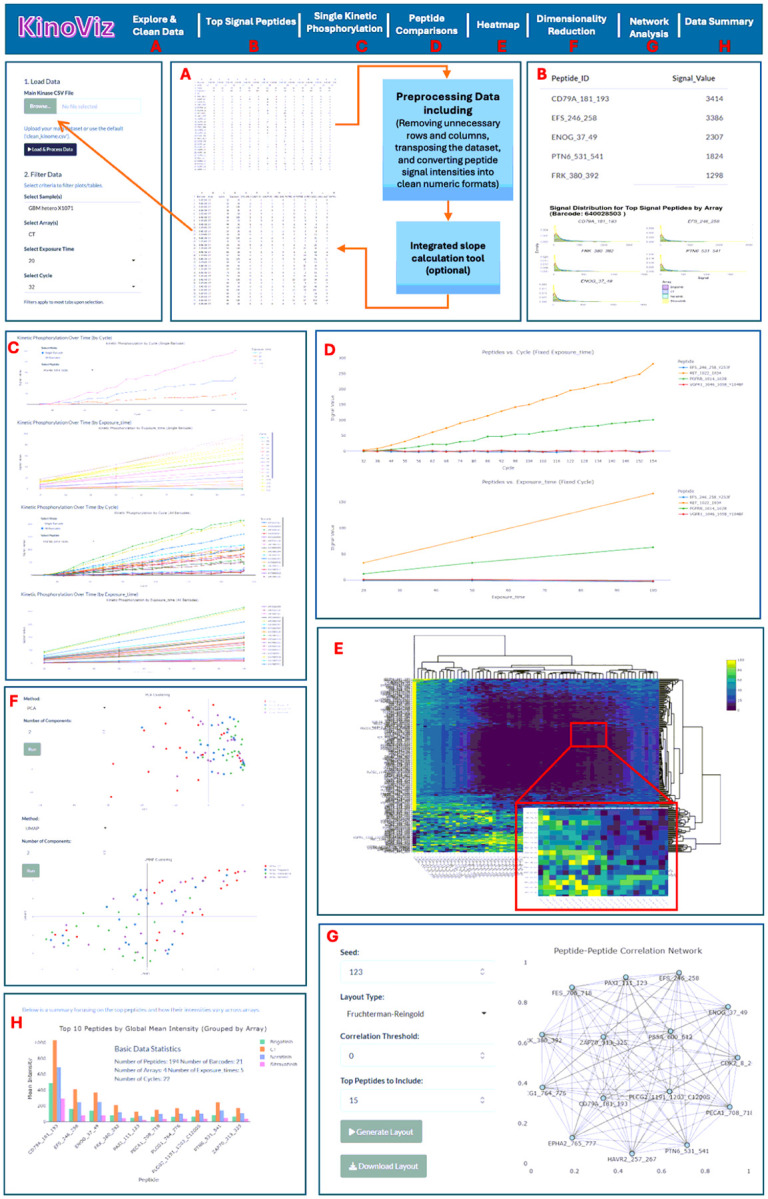
Overview of the KinoViz web application interface and visualization modules. (A) Data upload and preprocessing interface, featuring interactive AG Grid tables for data filtering and exploration. (B) Identification and ranking of peptides based on signal intensity, emphasizing peptides with the highest phosphorylation signals. (C) Visualization of kinetic phosphorylation patterns across multiple experimental cycles and exposure times. (D) Comparative phosphorylation intensity plots for selected peptides under different experimental conditions. (E) Heatmap illustrating hierarchical clustering patterns of peptide phosphorylation intensities across samples. (F) Dimensionality reduction analysis (principal component analysis, PCA) for exploration of global phosphorylation intensity patterns. (G) Peptide–peptide correlation network analysis demonstrating relationships and connectivity among peptide phosphorylation events. (H) Statistical summary module, including comparative bar charts highlighting the top phosphorylated peptides.

KinoViz is a comprehensive R Shiny web application that simplifies kinome array data analysis by streamlining complex workflows into an accessible interface. The application automates data normalization while providing interactive visualizations allowing researchers to extract biologically meaningful insights without requiring extensive computational expertise. KinoViz employs a systematic four-step workflow: 1) normalization, 2) peptide selection, 3) experimental condition definition, and 4) visualization (including phosphorylation levels, networks, heatmaps, and dimensional reductions). This structured approach converts raw datasets into interpretable results while preserving the integrity of the biological information (See Fig. 1 for an overview of the KinoViz workflow and functionalities).

### 3.1. Preprocessing Module: AG Grid Table Interface

The Preprocessing Module offers an interactive AG Grid Table, enabling users to explore and manipulate data dynamically. Users can observe kinetic parameters and navigate datasets through multi-peptide selection, filtering, and sorting. Curated data can be exported for external analysis (Fig. 1A).

Once a CSV file is uploaded, automated routines immediately clean the data by removing irrelevant rows and columns, transposing datasets, while ensuring peptide signal intensities are in consistent numerical formats, creating a structured dataset for downstream analyses. Additionally, the module includes a built-in slope calculation tool that determines phosphorylation rates based on changes in overall signal intensity, and kinetic differences.

### 3.2. Top Signal Peptides Module

This module offers an interactive way to explore peptides with the highest detected signal intensities linked to a selected barcode (i.e., PamChip). Users can choose the number of peptides to display, generating a table that lists peptide identifiers alongside their signal values. To enhance data interpretation, the module also features density plots that visualize the distribution of signal intensities across multiple arrays. These plots allow users to easily compare signal levels and assess variability among the highest signal peptides (Fig. 1B).

### 3.3. Kinetic Phosphorylation module

The Single Kinetic Phosphorylation module feature offers a two-mode selector via a two-way radio button: “Single Barcode” and “All Barcodes.” Each barcode is a unique identifier of the 4-well PamChip, with each well containing a peptide array. Therefore, a barcode and array number designation will uniquely identify a particular sample lysate (Supplemental Table 1). Sample comparisons can be made across both arrays and barcodes. When in “Single Barcode” mode, the user can select a peptide associated with a specific barcode. The application will create two plots of kinase kinetics. The first plot is a graph of changes in phosphorylation intensity over cycle numbers at a fixed (camera) exposure time (Fig. 1C). The second plot illustrates changes in phosphorylation intensity over camera exposure times at a fixed cycle number (Fig. 1C). In “All Barcodes” mode, the filtering is slightly altered. The user selects one or more peptides and specifies either an exposure time or a cycle number. The first plot is a graph of phosphorylation intensity data for all selected barcodes at the exposure time selected by the user, with cycle number on the x-axis (Fig. 1C). The second plot is of phosphorylation intensity data for all barcodes at the selected cycle number, with exposure time on the x-axis (Fig. 1C).

Similar to the Single Kinetic Phosphorylation module in Single Barcode mode, the Multiple Kinetic Phosphorylation module also generates two figures but allows simultaneous analysis of multiple peptides for a specific exposure time (peptides vs. cycle (fixed exposure time)) and specific cycle number (fixed cycle number). Both modules support analyses for entire peptide arrays as well as specific barcodes (Fig. 1D).

### 3.4. Heatmap Module

The Heatmap module provides a visual representation of phosphorylation intensities across multiple samples (barcodes) and peptides from all arrays. Users can tailor the heatmap to their needs by selecting a specific exposure time or cycle number. The data is structured as a matrix, with peptides arranged in rows and samples/barcodes/arrays in columns. To emphasize relative differences in phosphorylation levels, the data is normalized using z-scores. Hierarchical clustering helps identify patterns by grouping peptides based on their phosphorylation similarities. The interactive design, powered by *plotly* package, allows users to zoom in and access detailed insights through tooltips, making data exploration intuitive and dynamic (Fig. 1E).

### 3.5. Dimension Reduction Module

The Dimensionality Reduction module allows users to convert complex kinase, or peptide data, into an interpretable lower-dimensional space. Users can either choose Principal Component Analysis (PCA) [41], a linear dimensionality reduction technique that preserves global variance, or Uniform Manifold Approximation and Projection (UMAP)[42], a non-linear technique that keeps the local structure of the data and reveals more subtle datasets. After a method has been selected, the number of components must be specified (e.g., 2 for a 2D scatter plot, or 3 for a 3D visualization) in order to generate the projection. Each point on the scatter plot corresponds to a specific array, from a particular sample/barcode. In the presented data an array is specific to drug, or vehicle control treatment, while barcode is specific to PDX/tumor of origin (Fig. 1F).

### 3.6. Network Analysis Module

The Network Analysis module uses an interactive graphical representation of relationships among peptides to facilitate the identification of patterns or clusters of co-varying or potentially co-regulated peptides. In this representation, the nodes correspond to the individual peptides, and the edges represent a significant correlation or chosen measured metric between peptide signals. There are several parameters that can be customized that will allow for a more tailored analysis: Seed: Ensures that the network layout is consistent for reproducibility. *Layout Type*: Provides several force-directed algorithms, including Fruchterman-Reingold and Kamada-Kawai, to optimize the arrangement of the nodes. Correlation Threshold: Specifies the minimum correlation required to determine that two or more peptides are connected. *Selected Peptides* to Include: Limits the display of the network to show only the most highly correlated or top variable peptides (Fig. 1G).

### 3.7. Data Summary Module

The Data Summary module offers an overview of the experimental data that highlights critical parameters, including the total number of peptides, barcodes, arrays, exposure time and cycles. The module makes bar plots of the peptides ranked by global mean intensity, with the peptides grouped by array. This provides a way to quickly assess and compare intensity variability for peptides under experimental conditions and across comparative arrays.

## 4. Conclusion

We developed KinoViz as an easy-to-use, web-based platform that simplifies the analysis and interpretation of kinome array data using *ex vivo* drug response modeling as an example. With a suite of interactive and intuitive tools including: 1) kinetic phosphorylation profiles, 2) identification of altered peptides, 3) heatmaps, and 4) advanced visualization modules like network analysis and dimensionality reduction (PCA/UMAP), KinoViz helps researchers move from raw data to actionable insights more efficiently. Built to process complex datasets generated from PamStation kinomic arrays, KinoViz is specifically designed for researchers who may not have extensive bioinformatics expertise. The main limitation to the application is the lack of robust statistical testing into the visualizations. In addition, the application’s slope correction does differ from PamGene’s BioNavigator that may produce subtle differences compared to the commercial software. Nevertheless, KinoViz provides an accessible and generally, straightforward web application to identify differential peptide phosphorylation downstream of active kinases, explore phosphorylation dynamics, and visualize potential therapeutic targets, within a user-friendly interface. By facilitating the visualization and interpretation of high-throughput kinase activity assays, KinoViz allows better workflow efficiency which is critical in cancer drug discovery and other research. Looking ahead, we plan to expand KinoViz’s analytical capabilities to further support precision oncology and personalized medicine, solidifying its role as a vital resource for cancer research.

## Data Availability

The data shown in this manuscript is available through the KinoViz software and is included as the example data. KinoViz Shiny app is available at: https://willeycd.shinyapps.io/KinoViz.
